# The systemic oxidative stress index predicts clinical outcomes of esophageal squamous cell carcinoma receiving neoadjuvant immunochemotherapy

**DOI:** 10.3389/fimmu.2025.1535507

**Published:** 2025-01-31

**Authors:** Jifeng Feng, Liang Wang, Xun Yang, Qixun Chen

**Affiliations:** ^1^ Department of Thoracic Surgery, Zhejiang Cancer Hospital, Hangzhou Institute of Medicine (HIM), Chinese Academy of Sciences, Hangzhou, Zhejiang, China; ^2^ Key Laboratory Diagnosis and Treatment Technology on Thoracic Oncology, Zhejiang Cancer Hospital, Hangzhou, Zhejiang, China

**Keywords:** systemic oxidative stress, systemic oxidative stress index, esophageal squamous cell carcinoma, disease-free survival, overall survival, prognosis

## Abstract

**Background:**

Strong correlations have been shown between systemic oxidative stress (SOS) and the occurrence, metastasis, and prognosis of many types of cancers. It is yet unknown how SOS levels relate to the prognosis of esophageal squamous cell carcinoma (ESCC). The current research aims to explore the prognostic role of systemic oxidative stress index (SOSI) on ESCC receiving neoadjuvant immunochemotherapy (nICT).

**Methods:**

Retrospective recruitment was used to identify 224 nICT-treated ESCC patients. In order to determine the integrative score of SOSI, logistic regression analyses were utilized to screen independent risk variables, with disease-free survival (DFS) serving as the dependent variable. Given the non-linear relationship between SOSI and DFS, the best threshold was determined using a restricted cubic spline (RCS) model. Independent variable determination was executed using a cox regression analysis. For prognostic prediction, a risk categorization method based on recursive partitioning analysis (RPA) was also created.

**Results:**

Four SOS-related indicators, including albumin, creatinine, blood urea nitrogen, and direct bilirubin, were used to establish the SOSI. The ideal threshold of SOSI, shown by the non-linear relationship between DFS and SOSI (P<0.001), was used to compare between two groups. As a potential prognostic factor for those nICT-treated ESCC patients, SOSI showed a strong correlation with both DFS and overall survival (OS). Patients with low SOSI had better DFS (55.1% vs. 85.5%, P<0.001) and OS (72.6% vs. 79.1%, P=0.013). Then, a new staging that included TNM and SOSI based on RPA algorithms was produced. In terms of prognostication, the RPA model performed significantly better than TNM classification.

**Conclusion:**

SOSI is a simple and useful score based on available SOS-related indices. In ESCC receiving nICT, low SOSI is found to be an important factor of better prognosis.

## Highlights

- The prognostic impact of SOSI in ESCC remained unclear.
- SOSI was associated with prognosis in ESCC receiving nICT.
- First study to confirm the predictive value of SOSI in ESCC receiving nICT.

## Introduction

One of the most prevalent and aggressive malignant tumors worldwide, particularly in China, is esophageal cancer (EC), which primarily consists of two pathological subtypes: esophageal squamous cell carcinoma (ESCC) and esophageal adenocarcinoma (EAC) (1, 2). Neoadjuvant therapy, such as neoadjuvant chemoradiotherapy or chemotherapy followed by surgery has emerged as the current standard treatment mode for EC in light of the advancements in medicine, technology, and drug development in recent years (3, 4). Therefore, more and more appropriate and effective therapeutic methods are required to assess the long-term prognosis, as the current long-term survival for EC is still unsatisfactory. For locally advanced EC, neoadjuvant immunochemotherapy (nICT), a newly recognized therapeutic hotspot, has shown to be both safe and effective (5–7). However, the clinical outcomes, especially for the prognostic prediction, of nICT in EC require further verification.

It is well known that the most accurate predictor of cancer prognosis is thought to be the TNM system (8). Nevertheless, it has been established that tumorigenesis is a multi-stage, multi-step biological process. Therefore, the current TNM staging system is straightforward and ignores a number of important factors that could affect the cancer survival. Therefore, researchers continue to explore and develop more and more new prognostic indicators. An imbalance between free radicals and reactive metabolites is known as systematic oxidative stress (SOS), and it has been linked to the cancer development, progression, metastasis, and occurrence (9). Currently, numerous investigations have documented the significant role of reactive oxygen species (ROS) in cancers (10–12). In recent years, considerable evidence has demonstrated that several hematologic indices could reflect the status of SOS. An elevated SOS was linked to a sleep-deprived mice model that revealed substantial increases in blood urea nitrogen (BUN), creatinine (CRE), lactate dehydrogenase (LDH), and total bilirubin (TBIL) (13). Compared to critically ill polytrauma patients treated with antioxidant, additionally, the levels of TBIL, LDH, C-reactive protein (CRP), and albumin (ALB) were significant statistical changes in those without treatment (14). These indices have been proposed as potential SOS indicators. Therefore, an increasing number of researchers have employed the biological indices mentioned above to forecast the prognosis of various cancers (15–18).

Nevertheless, the correlation between SOS and the prognosis of EC remains unclear. Given the significant role that SOS played in the development of EC, we attempted to investigate the possible prognostic implications of SOS-related indices. The purpose of this study is to investigate, using a variety of biochemical indicators associated with SOS, the association between SOS and the ESCC prognosis. Additionally, on the basis of the aforementioned SOS related indices, a novel systematic oxidative stress index (SOSI) was developed. Moreover, the clinical prognostic superiority was ascertained by comparing the prognostic values of SOSI with other conventional indices. To compare the prognostic superiority, in addition, a novel staging based on SOSI and TNM was also developed.

## Methods

### Study design and patient selection

Participants in the current study included hematological indices and clinicopathologic data of ESCC patients who underwent radical resection after nICT between 2019 and 2021. The following criteria were used for inclusion: 1) confirmed ESCC by histopathology; 2) received nICT before radical surgery; 3) received radical resection without any evidence of distant metastasis; 4) contained comprehensive clinicopathologic data and follow-up; 5) excluded any infectious, immune, inflammatory, and hematological diseases. Patients were excluded if they had: 1) other pathological types; 2) non-radical surgery; 3) other antitumor therapies (in addition to nICT); 4) multiple primary cancers in addition to EC (previous or concurrent); 5) anti-inflammatory drugs prior to radical surgery; 6) hepatorenal dysfunction, metabolic diseases, or cardiovascular disease. The AJCC/UICC TNM classification system (8th edition) was applied for this research (19). This study, which complied with the Helsinki Declaration, was approved by the Ethics Committee (IRB-2020-183).

### Therapeutic process and follow-up

Two cycles of nICT before radical surgery were administered to each patient every 21 days. On day 1, an immunological drug (200mg of camrelizumab, tislelizumab, or sintilimab; or 2mg/kg of pembrolizumab; or 3mg/kg of nivolumab) was infusion. Then, carboplatin (on day 1: 5 mg/ml/min in area under the curve) and albumin-bound paclitaxel (on days 1 and 8: 120 mg/m^2^) were part of the therapeutic regimen. Radical surgery in the McKeown or Ivor Lewis procedure was often arranged to take place 4-6 weeks following the result of the last cycle of nICT (20). Regarding adjuvant treatment after nICT, no consensus has been formed as of yet. Adjuvant immunotherapy may be beneficial for patients following neoadjuvant therapy, as per the findings of the CheckMate 577 study (21). Therefore, adjuvant immunotherapy was carried out following radical surgical resection in the current study, but not mandatory, particularly for those with ypN+ and/or ypT3-4 staging in postoperative pathological results. December 2022 is the last day of the follow-up period.

### Data collection and definition

Retrospectively gathered and organized data from our electronic medical records including clinical features, and different pretreatment hematological indices. Hematological indices, such as TBIL, ALB, CRP, LDH, BUN, CRE, direct bilirubin (DBIL), uric acid (UA), neutrophil (NEU), platelet (PLT), and lymphocyte (LYM), were obtained within 1 week before nICT. NEU divided by LYM and PLT divided by LYM, respectively, were the definitions of the neutrophil to lymphocyte ratio (NLR) and platelet to lymphocyte ratio (PLR) (22). The following formula was used to generate the systemic immune-inflammation index (SII): PLT × NEU/LYM (22).

### Statistical analysis

Logistics analyses, both univariate and multivariate, were used to identify the independent biochemical variables of disease-free survival (DFS). The systematic oxidative stress index (SOSI) was then calculated using four ideal variables, including CRE, ALB, DBIL, and BUN. SOSI and other hematological biochemical indices were compared, and their discrimination, clinical relevance and areas under the receiver operator characteristic (ROC) curve (AUCs) were assessed using calibration curves (CCs), decision curve analyses (DCAs) and ROCs. To predict overall survival (OS) and DFS, Cox regression analyses were conducted using hazard ratios (HRs) and 95% confidence intervals (CIs). For prognostication and stratification, an SOS based model for risk stratification was created by recursive partitioning analysis (RPA). ROCs and DCAs were utilized to assess the prognostic efficacy of the current SOSI-based model. This investigation was conducted by using SPSS 20.0, Medcalc 15.2.2, and R 4.1.2 software. Statistical significance was indicated by P <0.05.

## Results

### Creation of SOSI

SOS was created using the techniques in previous research (15–18). In **Supplementary Figure S1**, the SOSI’s process diagram is displayed. Possible variables were initially chosen using univariate logistic analysis in order to assess the predictive importance of the SOS-related metrics. Ultimately, multivariate analysis was performed on variables from the univariate logistic analysis that had a P value less than 0.1. The continuous variables CRE, DBIL, ALB, and BUN were found to be significant independent predictors based on the studies. With the help of the logistic regression equation, an integrative score known as SOSI was subsequently determined as follows: SOSI = -0.064 × CRE + 0.679 × DBIL + 0.635 × BUN - 1.781 × ALB.

### Comparisons between SOSI and other hematological indices

**Figure 1:**
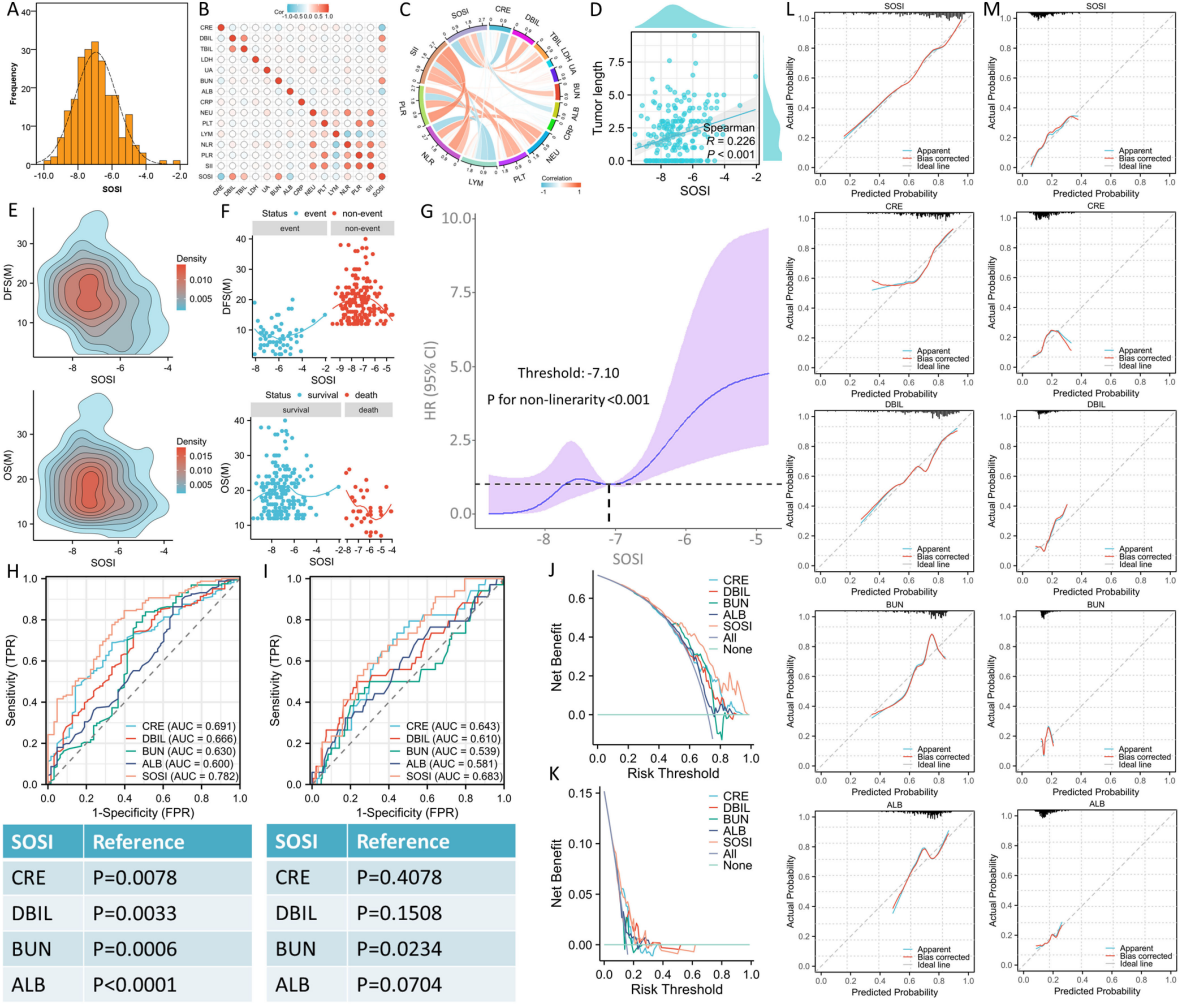
The distribution of SOSI, the selection of optimal cut-off value and the comparison with other indicators. The values of SOSI are normally distributed **(A)**. The correlation **(B)** and chord **(C)** diagrams of hematological indices. The correlation between SOSI and tumor length **(D)**. The density profile between SOSI and prognosis **(E)**. The link between SOSI and prognosis implies a non-linear relationship between them **(F)**. A RCS model was used to establish the optimal SOSI threshold **(G)**. ROC curve analyses indicated the maximum AUC in SOSI (0.782 for DFS and 0.683 for OS), signifying the highest prognostic ability of SOSI in DFS **(H)** and OS **(I)**. Better SOSI prediction values in DFS **(J)** and OS **(K)** were also shown by the DCA curves than by the other indices. The calibration curves of SOSI displayed a high degree of agreement between actual observation and prediction when compared to other indices in DFS **(L)** and OS **(M)**.

The mean value of SOSI was -6.94 ± 1.22. In **Figure 1A**, the SOSI distribution is displayed. The correlation and chord diagrams of all hematological indices, including SOSI, are shown in **Figures 1B, C**. As shown in **Figure 1D**, there was a positive connection (r=0.228, P<0.001) between SOSI and tumor length. The link between SOSI and DFS/OS is depicted in **Figures 1E, F**, which implies a non-linear relationship between them. Because of the non-linear relation, a RCS model was utilized to establish the optimal SOSI threshold (**Figure 1G**). Then patients were divided into two groups for further analysis. SOSI and other hematological indices were examined for prognostic values in order to assess the SOSI’s superiority. Based on ROC curve analysis, the maximum AUC in SOSI was discovered, signifying the highest prognostic ability of SOSI (**Figures 1H, I**). Better SOSI prediction values in OS and DFS were also shown by the DCA curves than by the other indices (**Figures 1J, K**). Compared to other hematological indices, the SOSI showed a large positive net benefit from the risk of mortality, demonstrating its tremendous clinical practical usefulness in predicting DFS and OS. The CC of SOSI displayed a high degree of agreement between actual observation and prediction when compared to other indices (**Figures 1L, M**).

### Patient characteristics grouped by SOSI

The mean age was 63.4 ± 6.7 years (range: 47-75 years). The median follow-up time was 18 months (range: 7-40 months). **Table 1** displays the clinical features categorized by SOSI. Higher SOSI patients tended to have more advanced ypT staging (P=0.046), more vascular invasion (P=0.016), more perineural invasion (P=0.044), and longer tumor lengths (P=0.038). In terms of other hematological indices, additionally, patients with high SOSI tended to have higher TBIL (P<0.001), DBIL (P<0.001), BUN (P<0.001), and NLR (P=0.031), and lower ALB (P<0.001) and CRE (P<0.001).

**Table 1:** Patient characteristics grouped by SOSI in ESCC receiving NICT.

	Low SOSI (n=110)	High SOSI (n=114)	P-value
Clinical characteristics			
Age (mean ± SD, years)	63.5 ± 6.9	63.2 ± 6.5	0.748
Sex (female/male, n, %)	7(6.4)/103(93.6)	14(12.3)/100(87.7)	0.129
Smoking history (yes/no, n, %)	82(74.5)/28(25.5)	77(67.5)/37(32.5)	0.248
Drinking history (yes/no, n, %)	73(66.4)/37(33.6)	80(70.2)/34(29.8)	0.540
BMI (mean ± SD, Kg/m^2^)	21.6 ± 2.0	21.8 ± 2.0	0.457
Tumor location (n, %)			0.849
upper	11 (10.0)	9 (7.9)	
middle	65 (59.1)	70 (61.4)	
lower	34 (30.9)	35 (30.7)	
Surgical method (MK/IL, n, %)	93(84.5)/17(15.5)	99(86.8)/15(13.2)	0.623
Differentiation (n, %)			0.504
well	21 (19.1)	29 (25.4)	
moderate	52 (47.3)	48 (42.1)	
poor	37 (33.6)	37 (32.5)	
Vessel invasion (yes/no, n, %)	7(6.4)/103(93.6)	19(16.7)/95(83.3)	0.016
Perineural invasion (yes/no, n, %)	13(11.8)/97(88.2)	25(21.9)/89(78.1)	0.044
Tumor length (mean ± SD, cm)	1.71 ± 1.67	2.22 ± 1.95	0.038
PCR (yes/no, n, %)	36(32.7)/74(67.3)	32(28.1)/82(71.9)	0.449
ypT stage (n, %)			0.046
T0	36 (32.7)	32 (28.1)	
T1-2	42 (38.2)	31 (27.2)	
T3-4a	32 (29.1)	51 (44.7)	
ypN stage (positive/negative, n, %)	39(35.5)/71(64.5)	53(46.5)/61(53.5)	0.093
ypTNM stage (n,%)			0.376
0	36 (32.7)	32 (28.1)	
I-II	33 (30.0)	29 (25.4)	
III-IVA	41 (37.3)	53 (46.5)	
Hematological variables			
CRE (mean ± SD, μmol/L)	85.99 ± 7.07	75.46 ± 8.72	<0.001
DBIL (mean ± SD, μmol/L)	3.68 ± 0.61	4.40 ± 0.94	<0.001
TBIL (mean ± SD, μmol/L)	8.08 ± 1.75	9.22 ± 2.23	<0.001
LDH (mean ± SD, U/L)	188.98 ± 64.1	188.33 ± 58.8	0.937
UA (mean ± SD, μmol/L)	237.79 ± 49.6	249.11 ± 52.7	0.099
BUN (mean ± SD, mmol/L)	4.02 ± 0.42	4.70 ± 0.94	<0.001
ALB (mean ± SD, g/dL)	4.18 ± 0.19	4.01 ± 0.25	<0.001
CRP (mean ± SD, mg/L)	3.29 ± 2.59	3.11 ± 2.24	0.578
NEU (mean ± SD, 10^9^/L)	4.35 ± 1.00	4.77 ± 1.36	0.008
PLT (mean ± SD, 10^9^/L)	222.25 ± 68.0	221.18 ± 61.1	0.901
LYM (mean ± SD, 10^9^/L)	1.49 ± 0.40	1.47 ± 0.44	0.834
NLR (mean ± SD)	3.11 ± 1.04	3.44 ± 1.23	0.031
PLR (mean ± SD)	157.14 ± 55.9	158.43 ± 51.6	0.858
SII (mean ± SD)	683.76 ± 298.4	765.94 ± 373.4	0.071

SOSI, systematic oxidative stress index; ESCC, esophageal squamous cell carcinoma; NICT, neoadjuvant immunochemotherapy; SD, standard deviation; BMI, body mass index; PCR, pathological complete response; TNM, tumor node metastasis; CRE, creatinine; DBIL, direct bilirubin; TBIL, total bilirubin; LDH, lactate dehydrogenase; UA, uric acid; BUN, blood urea nitrogen; ALB, albumin; CRP, C-reactive protein; NEU, neutrophil; PLT, platelet; LYM, lymphocyte; NLR, neutrophil to lymphocyte ratio; PLR, platelet to lymphocyte ratio; SII, systemic immune-inflammation index.

### Predictors to DFS and OS

**Figure 2:**
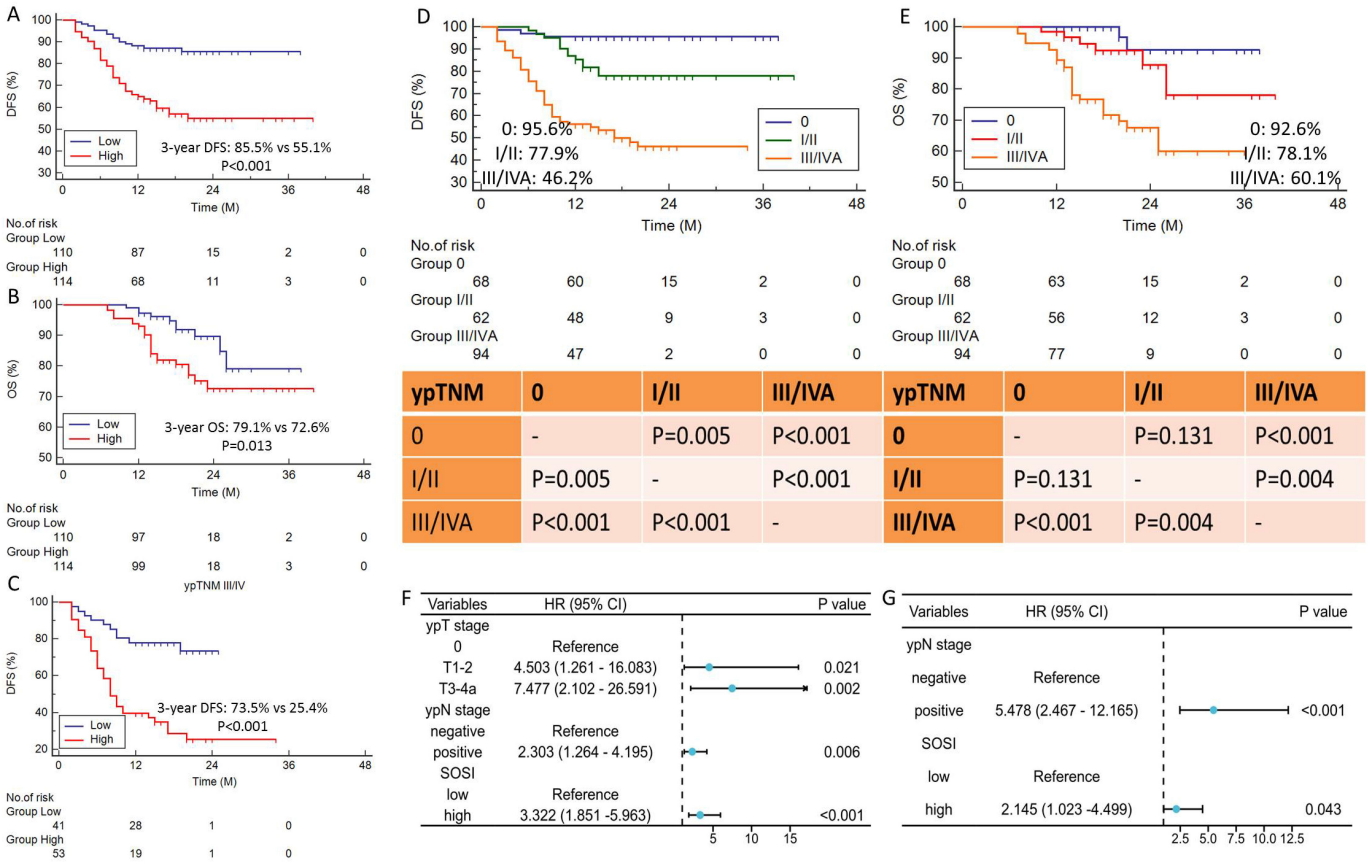
Survival curves and prognostic forest plot. DFS **(A)** and OS **(B)** grouped by SOSI. Subgroup analysis of SOSI in ypTNM III/IV stage **(C)**. High SOSI had lower 3-year DFS (55.1% vs. 85.5%, P<0.001) and OS (72.6% vs. 79.1%, P=0.013). The DFS **(D)** and OS **(E)** are categorized by ypTNM stage. Forest plots indicated the results of multivariate cox regression analyses in DFS **(F)** and OS **(G)**. SOSI was an independent predictor of DFS (P=0.040) and OS (P=0.015).

Compared to patients with low SOSI, those with high SOSI had lower 3-year DFS (55.1% vs. 85.5%, P<0.001; **Figure 2A**) and OS (72.6% vs. 79.1%, P=0.013; **Figure 2B**). Subgroup analyses were also performed in SOSI based on ypTNM. In the ypTNM III/IVA subgroup, there is a striking difference in DFS (73.5% vs. 23.4%, P<0.001, **Figure 2C**) between the low and high SOSI groups. There was no significant difference between the other groups. The DFS and OS are categorized by ypTNM in **Figures 2D, E**. Nevertheless, there was no statistically significant difference between ypTNM 0 and I/II, particularly for OS (P=0.131). **Table 2** displays the results of the univariate Cox regression analyses for DFS and OS. SOSI was an independent predictor of DFS (HR =3.322, P=0.040; **Figure 2F**) and OS (HR=2.145, P=0.015; **Figure 2G**), according to the multivariate analyses. According to the findings, compared to the low SOSI group, a high SOSI group had a 3.322-fold and 2.145-fold higher risk of death and recurrence in the current research, respectively.

**Table 2:** Univariate analyses of prognostic factors associated with DFS and OS in ESCC receiving NICT.

	DFS		OS	
	HR (95% CI)	P-value	HR (95% CI)	P-value
Age (years, >70 vs. ≤70)	0.576 (0.284-1.167)	0.126	0.440 (0.155-1.250)	0.123
Sex (male vs. female)	0.977 (0.421-2.267)	0.957	0.929 (0.284-3.041)	0.903
Smoking history (yes vs. no)	1.106 (0.634-1.930)	0.723	0.723 (0.351-1.487)	0.378
Drinking history (yes vs. no)	1.618 (0.906-2.890)	0.104	1.893 (0.824-4.349)	0.132
BMI (Kg/m^2^, >20 vs. ≤20)	0.658 (0.373-1.161)	0.148	0.609 (0.290-1.276)	0.189
Tumor location				
upper	reference		reference	
middle	0.547 (0.253-1.185)	0.126	0.445 (0.162-1.217)	0.115
lower	0.758 (0.337-1.703)	0.502	0.641 (0.228-1.803)	0.400
Surgical method (IL vs. MK)	0.803 (0.382-1.685)	0.561	0.492 (0.150-1.610)	0.241
Differentiation				
well	reference		reference	
moderate	1.816 (0.864-3.815)	0.115	2.143 (0.808-5.687)	0.126
poor	1.726 (0.798-3.730)	0.165	0.992 (0.324-3.036)	0.989
Vessel invasion (yes vs. no)	3.219 (1.822-5.689)	<0.001	1.756 (0.727-4.243)	0.211
Perineural invasion (yes vs. no)	2.324 (1.345-4.018)	0.003	1.834 (0.855-3.931)	0.119
Tumor length (cm, >3 vs. ≤3)	3.392 (2.060-5.586)	<0.001	2.771 (1.395-5.507)	0.004
ypT stage				
T0	reference		reference	
T1-2	6.416 (1.898-21.687)	0.003	5.440 (1.205-24.566)	0.028
T3-4a	14.649 (4.533-47.343)	<0.001	10.110 (2.365-43.213)	0.002
ypN stage (positive vs. negative)	4.766 (2.754-8.248)	<0.001	5.839 (2.632-12.953)	<0.001
ypTNM stage				
0	reference		reference	
I-II	4.851 (1.382-17.024)	0.014	3.339 (0.673-16.556)	0.140
III-IVa	15.332 (4.767-49.311)	<0.001	11.364 (2.690-48.013)	0.001
PCR (yes vs. no)	0.096 (0.030-0.307)	<0.001	0.128 (0.031-0.536)	0.005
SOSI (high vs. low)	3.640 (2.037-6.503)	<0.001	2.449 (1.171-5.122)	0.017

ESCC, esophageal squamous cell carcinoma; DFS, disease-free survival; OS, overall survival; NICT, neoadjuvant immunochemotherapy; SOSI, systematic oxidative stress index; HR, hazard ratio; CI, confidence interval; BMI, body mass index; MK, McKeown; IL, Ivor Lewis; TNM, tumor node metastasis; PCR, pathological complete response.

### Stratification model established based on SOSI

By employing the RPA algorithm, an SOSI-based classification was created (**Supplementary Figure S2A**). With considerably varying DFS and OS, the RPA model split all patients into three groups. The RPA based classification showed better stratification in both OS and DFS compared to the ypTNM staging, particularly for RPA I and II (**Supplementary Figures S2B, C**). Additionally, the prognostic efficacy of the SOSI-based RPA model was assessed in comparison to the ypTNM staging. The SOSI based RPA model outperformed the ypTNM staging in terms of prediction accuracy, as indicated by the ROC curves (**Supplementary Figures S2D, E**). Parallel to this, the higher prognostication accuracy of the RPA model was also confirmed by the DCA curves (**Supplementary Figures S2F, G**). Sankey diagrams were used to analyze the relationship between SOSI and clinical outcomes (**Supplementary Figure S2H**).

## Discussion

Neoadjuvant therapy has achieved positive results in various gastrointestinal tumors, including gastric, esophageal and gastro-esophageal junction cancers (21, 23). Postoperative adjuvant therapy has positive effect on these gastroesophageal related cancers (21, 24). For ESCC patients receiving nICT, to our knowledge, this research is the first to investigate the relevance of SOS-related indicators for prognosis and to develop a predictive model using SOSI. This study developed a comprehensive index (SOSI) to predict the prognosis of ESCC based on DBIL, CRE, ALB, and BUN. We investigated the relationship between SOSI and clinicopathological variables of ESCC. The SOSI was related to larger tumor size, vessel invasion, perineural invasion, and advanced ypT staging. Recent study revealed that pCR is a valid predictor for survival when using nICT (25). In the current study, there is no difference in pCR or ypTNM stage between the low and high SOSI groups. Cox regression analyses, both univariate and multivariate, revealed that SOSI was a possible prognostic factor in ESCC in both DFS and OS. Subsequent examination of SOSI’s prognostic utility demonstrated that patients with lower SOSI had longer 3-year DFS and OS. By employing the RPA algorithm, a new staging system based on SOSI was created. These findings suggested that SOSI played a significant role in prognosis of ESCC receiving nICT.

At present, various studies also use relevant SOS related indicators to develop matching prediction models and predict prognosis in a number of cancers (15–18). Using training and validation cohorts of 1583 patients with breast cancer, the relationship between the cancer prognosis and a novel systematic oxidative stress score (SOS based on LDH, TBIL, CRE, ALB, and BUN) was investigated. The results showed that SOS was a reliable indicator of the prognosis, which were additionally validated by a nomogram model based on SOS and other clinical factors (15). An additional investigation involving 1422 colorectal cancer patients (training: 1022 and validation: 400) revealed a substantial correlation between survival and the colorectal cancer-integrated oxidative stress score (CIOSS), which was determined by combining the available SOS related indices (ALB, DBIL, and BUN). Compared to TNM stage, the authors confirmed better predictive performance of CIOSS in colorectal cancer (16). Results were also confirmed the prognostic values of the integrated oxidative stress score (IOSS) in gastric cancer and the systematic oxidative stress indices (SOSI) in upper urinary tract urothelial carcinoma (17, 18).

Although SOS encourages the development of cancer, it is still unknown how SOS levels relate to the prognosis of cancer. Recent research indicated that the progression associated hub SOS genes were confirmed to be significantly related to the advancement of cancer (26). Therefore, early assessment of SOS can ameliorate the clinical outcomes for cancer patients. According to another study, metastatic cancer patients who received chemotherapy had SOS genes including NQO1 and PON1 as notable predictors of their prognosis. Additionally, genetic variations connected to SOS may help optimize tailored chemotherapy in clinical practice (27). Currently, the survival rate of high-risk groups was found to be lower than that of low-risk groups according to an SOS-related gene model, which provides new information about the possible use of the gene model in ESCC (28).

It is still unknown how the SOS-related hematological indices and the prognosis of ESCC are related, despite the fact that SOSI was substantially associated with prognosis in ESCC. CRE is a standard biomarker for assessing renal function. The human body produces endogenous CRE as a byproduct of muscle metabolism. Due to their correlation with renal dysfunction and the advancement of cancer, CRE levels have been linked in a number of studies to a poor prognosis in various cancers (29, 30). The primary byproduct of protein metabolism in the human body is BUN. High BUN levels reflect the function of many body systems and are associated with poor renal function, dehydration, and acute hemodynamic alterations (31). Consequently, the SOS status may be reflected in CRE and BUN. Bilirubin is a byproduct of heme metabolism and, despite its link with cancer prognosis, may have anticancer effects due to its antioxidant properties (32, 33). An essential protein made by the liver, ALB can provide information about an individual’s inflammatory response and nutritional status. ALB also possesses enzymatic activity and antioxidant properties. In patients with a variety of malignancies, including ESCC, higher ALB levels are linked to prolonged survival (34, 35).

To our knowledge, this investigation covered all published biochemical indices linked to SOS, such as BUN, LDH, CRP, TBIL, CRE, DBIL, UA, and ALB. We determined four independent SOS related biochemical variables (DBIL, BUN, CRE, and ALB) to compute SOSI after performing univariate and multivariate analyses. The association between SOS-related parameters and the prognosis of ESCC was first documented in this research, which also built a prediction model with an AUC of 0.683 for OS and 0.782 for DFS. For patients in SOS status, SOSI can offer sufficient prognostic information. It’s worth noting that there is no difference in pCR or ypTNM stage between the low and high SOSI groups. Nevertheless, the results of the study showed that there is a difference in survival between the two groups, indicating the influence of SOSI on prognosis may be independent of pCR or ypTNM stage. This further indicates that the recommended RPA model has certain clinical significance. Our findings suggested that SOSI has better stratification for patients with more advanced stages, indicating SOSI has heightened sensitivity for predicting recurrence in poor responders. By identifying individuals who have poor outcomes, this study may enable clinicians to treat high-risk patients more aggressively and to follow up with patients more frequently after radical surgery. In addition, the findings are helpful for further research on the connection between SOSI and cancer prognosis, and the results serve as a resource for the creation of SOSI therapeutic targets.

It is important to take into account a number of limitations on the current study. First off, just a small number of patients were included in this retrospective single-center analysis. There may be some gaps in data quality and integrity, and weaknesses in control variables that may lead to some bias. Further enrichment of the results would require more and more well-designed, prospective, multicenter investigations involving a larger number of ESCC cases. Secondly, the follow-up time for the current study is short, not all patients in our research have reached the 3-year point. With such limited long-term follow-up, the evidence for evaluating 3-year OS and/or DFS appears insufficient. Thirdly, it remains important to remember that different immunotherapy regimens can result in different outcomes. The evaluation of prognostic variables did not completely include the various postoperative therapies. Fourthly, even though the patients were selected based on strict inclusion and exclusion criteria, the results of the SOSI could still be affected by a variety of circumstances because it (combined with CRE, DBIL, BUN, and ALB) is derived from peripheral blood. Finally, there is a lack of studies on the mechanism of SOSI on the prognosis of nICT in ESCC. Further elucidating the mechanism is of great significance for predicting the prognosis of nICT in those with ESCC by SOSI. To validate the current findings, larger-scale clinical studies with more clinicopathological indices are also required.

## Conclusion

SOSI is a simple and useful predictor based on procurable SOS related indices, comprising CRE, ALB, DBIL, and BUN. It is found that in ESCC receiving nICT, low SOSI is a strong predictor of a better prognosis.

## Data Availability

The original contributions presented in the study are included in the article/**Supplementary Material**. Further inquiries can be directed to the corresponding author.

## Supplementary material

Supplementary Figure 1 The SOSI process diagram. Possible variables were chosen using univariate logistic analysis in order to assess the predictive importance of SOS-related metrics. Multivariate analysis was performed on variables from the univariate analysis that had a P value<0.1. The continuous variables CRE, DBIL, ALB, and BUN were found to be significant independent predictors based on the studies.

Supplementary Figure 2 RPA model established and compared. By employing the RPA-based algorithm, an SOSI-based classification was created **(A)**. The RPA based classification showed better stratification in DFS **(B)** and OS **(C)** compared to ypTNM staging. The SOSI based RPA model outperformed the ypTNM staging in terms of prediction accuracy, as indicated by ROC curves in DFS **(D)** and OS **(E)**. The higher prognostication accuracy of the RPA model was also confirmed by the DCA curves in DFS **(F)** and OS **(G)**. Sankey diagrams were used to analyze the relationship between SOSI and clinical outcomes **(H)**.
