# Supplementary figures and images for: The systemic oxidative stress index predicts clinical outcomes of esophageal squamous cell carcinoma receiving neoadjuvant immunochemotherapy
Source: Front Immunol. 2025 Jan 31;16:1535507. doi: 10.3389/fimmu.2025.1535507 (PMC11825508; doi:10.3389/fimmu.2025.1535507)

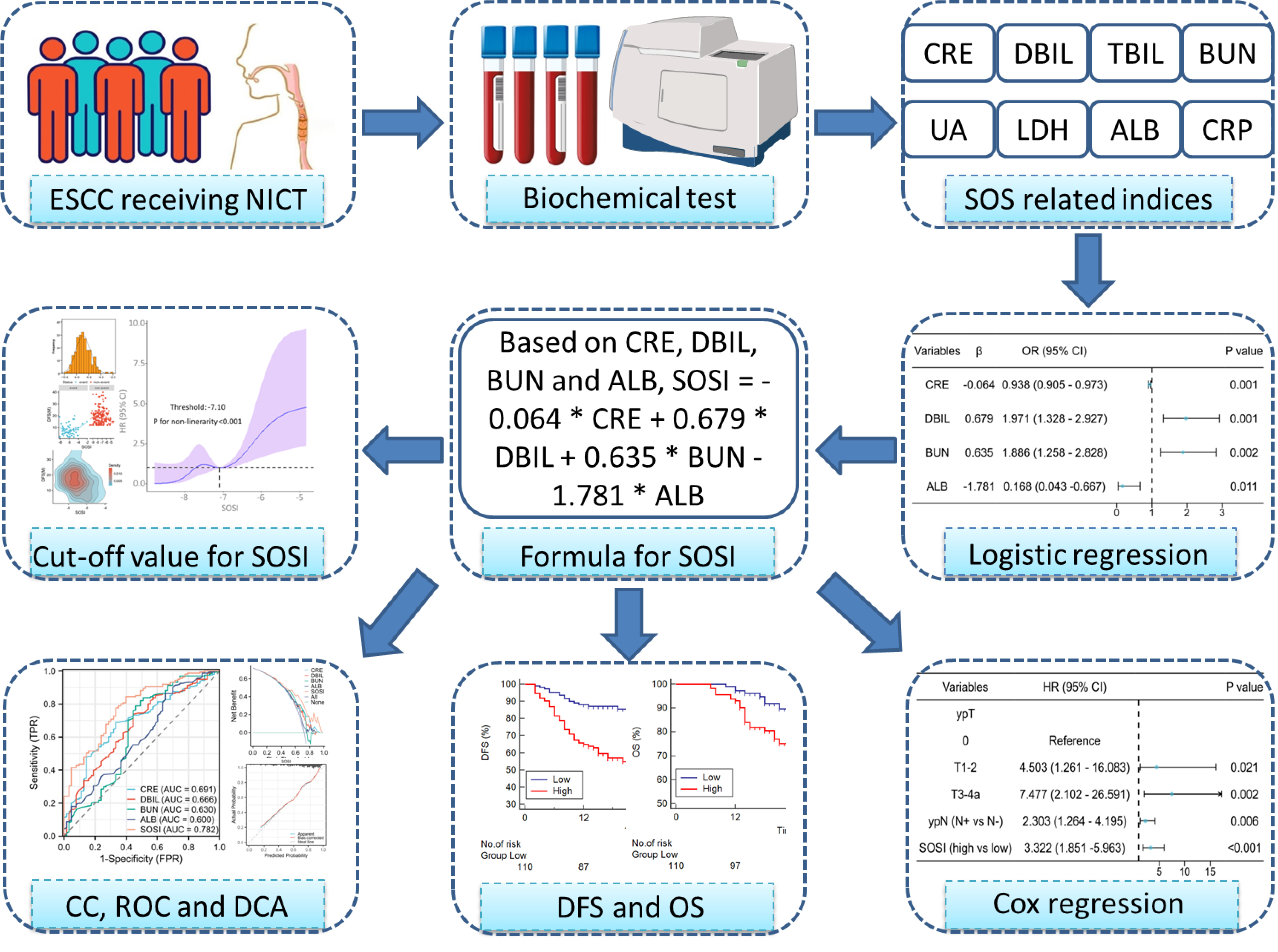

Supplement: Supplementary Figure 1 — The SOSI process diagram. Possible variables were chosen using univariate logistic analysis in order to assess the predictive importance of SOS-related metrics. Multivariate analysis was performed on variables from the univariate analysis that had a P value<0.1. The continuous variables CRE, DBIL, ALB, and BUN were found to be significant independent predictors based on the studies. [file Image1.jpeg]

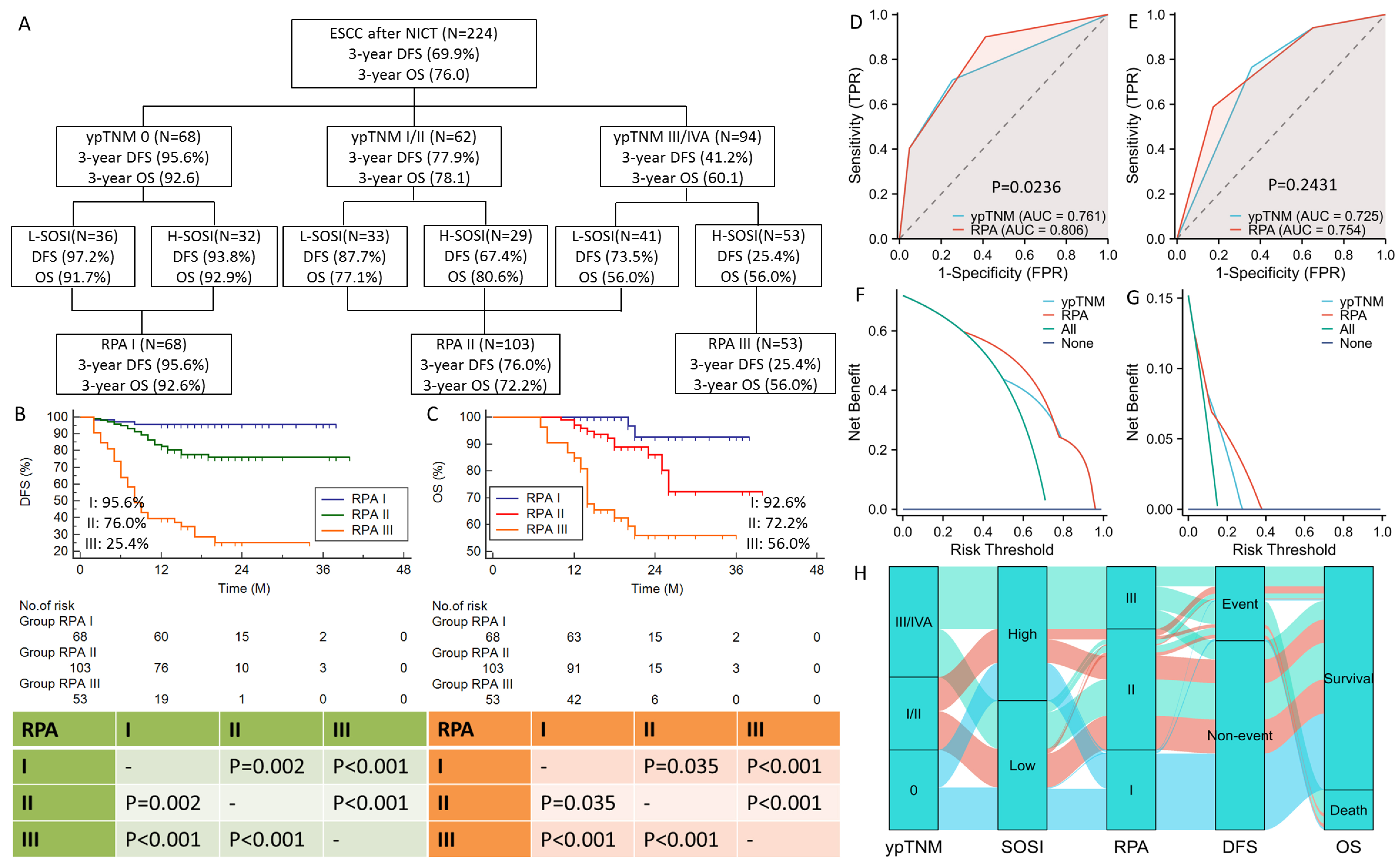

Supplement: Supplementary Figure 2 — RPA model established and compared. By employing the RPA-based algorithm, an SOSI-based classification was created (A). The RPA based classification showed better stratification in DFS (B) and OS (C) compared to ypTNM staging. The SOSI based RPA model outperformed the ypTNM staging in terms of prediction accuracy, as indicated by ROC curves in DFS (D) and OS (E). The higher prognostication accuracy of the RPA model was also confirmed by the DCA curves in DFS (F) and OS (G). Sankey diagrams were used to analyze the relationship between SOSI and clinical outcomes (H). [file Image2.jpeg]
